# Determining Whether Low Protein Intake (<1.0 g/kg) Is a Risk Factor for Malnutrition in Patients with Cirrhosis

**DOI:** 10.3390/jcm10102164

**Published:** 2021-05-17

**Authors:** Jin-Hwa Park, Minkoo Kang, Dae-Won Jun, Mimi Kim, Joo-Hee Kwak, Bo-kyeong Kang

**Affiliations:** 1 Department of Internal Medicine, Asan Medical Center, Ulsan University College of Medicine, Seoul 44610, Korea; pjh6718@hanmail.net; 2 Department of Internal Medicine, Hanyang University School of Medicine, Seoul 04763, Korea; kmg0508@gmail.com (M.K.); noshin@hanyang.ac.kr (D.-W.J.); jh_doc@hanmail.net (J.-H.K.); 3 Department of Radiology, Hanyang University School of Medicine, Seoul 04763, Korea; bluefish010@naver.com

**Keywords:** malnutrition, liver cirrhosis, protein

## Abstract

Background: The prevalence of malnutrition in patients with cirrhosis is considerably high. Body mass index (BMI) is a well-known risk factor for malnutrition, but the other risk factors are unknown. We investigated the prevalence of malnutrition and its risk factors in patients with cirrhosis. Methods: In total, 361 patients with cirrhosis were enrolled. Muscle quality and quantity were retrospectively assessed using the grip strength test and bioelectrical impedance analysis. Subjective global assessment (SGA) of malnutrition and dietary intake assessments were performed by a clinical dietician. Results: The prevalence rates of sarcopenia, malnutrition assessed by SGA, and inadequate energy intake were 22.7%, 13.6%, and 27.5%, respectively. The prevalence of malnutrition evaluated using any of the assessment methods was 46.3%, and no significant difference was observed according to liver disease etiology. The prevalence of malnutrition increased with the increasing disease severity (*p* = 0.034) and decreasing BMI (*p* = 0.007). The prevalence of malnutrition was 64.4% in patients with protein intake <1.0 g/kg. Low protein intake, Child–Pugh C grade, older age, and low BMI were independent risk factors for malnutrition in multivariate analysis. Conclusions: Low protein intake (<1.0 g/kg) is an independent risk factor for malnutrition in patients with cirrhosis.

## 1. Introduction

Malnutrition is one of the most common complications of cirrhosis and is associated with high mortality, high prevalence of infection, and portal hypertension-related complications, such as hepatic encephalopathy and ascites [1,2,3,4]. Nutritional assessments and monitoring are essential for patients with cirrhosis, and several tools for evaluating malnutrition have been proposed [5,6]. Several assessment tools are currently used to assess for malnutrition in patients with liver disease [7,8]; previous studies have reported a wide range of variability in the prevalence rate of malnutrition, from 5% to 99%, depending on the assessment tools used [3,9,10,11].

Recently, the European Association for the Study of the Liver proposed practice guidelines on nutrition in chronic liver disease [8]. Body mass index (BMI) and disease severity have been suggested as the most important risk factors for malnutrition, but the other risk factors are unknown.

Decreased protein intake is also an important risk factor for malnutrition [12]. The recommended daily protein intake in normal people is 0.83 g/kg [13], while in chronic liver disease patients it is 1.2–1.5 g/kg [5]. Patients with chronic liver disease are recommended to consume 1.5 times more protein than that consumed by normal individuals. Patients with chronic liver disease experience protein deficiency and have a high incidence of malnutrition due to the following reasons: reduced diet, indigestion, malabsorption (fat malabsorption, vitamin malabsorption, bacterial overgrowth, and portal hypertensive enteropathy), kidney-related diseases, and metabolic abnormalities [14]. The recommended protein intake is based on the minimum protein requirement to maintain the nitrogen balance. Therefore, patients with liver disease should consume 1.2–1.5 g/kg/day of protein. The protein intake should be 1.5 times higher than the usual intake to prevent sarcopenia, which can lead to worse clinical outcomes [15]. 

A cutoff protein value of 1.2–1.5 g/kg/day was reported in a previous study involving patients with cirrhosis who consumed a high-protein diet. The study also showed that patients with cirrhosis should consume up to 1.8 g/kg of protein [16]. However, a protein intake of 0.8 g/kg/day is required to achieve nitrogen balance in patients with alcoholic liver cirrhosis (LC) [17], and there is a lack of accurate evidence to show that 1.5 times higher protein intake than the usual intake can achieve nitrogen balance. Moreover, studies on the status of protein intake and adequate protein intake in patients with chronic liver disease are limited.

Previous studies on malnutrition and protein intake in patients with chronic liver disease have been conducted. In previous studies, the protein intake in patients with chronic liver disease was 1.16–1.31 g/kg [18,19,20]. However, the number of studies targeting all patients with chronic liver disease is relatively small, and the number of studies reporting the appropriate protein intake according to the severity of liver disease and various causes is limited.

Hence, we aimed to investigate the prevalence of malnutrition using various methods. In addition, we aimed to determinate whether low protein intake (<1.0 g/kg) is a risk factor for malnutrition in patients with cirrhosis.

## 2. Materials and Methods

### 2.1. Study Design

**Figure 1:**
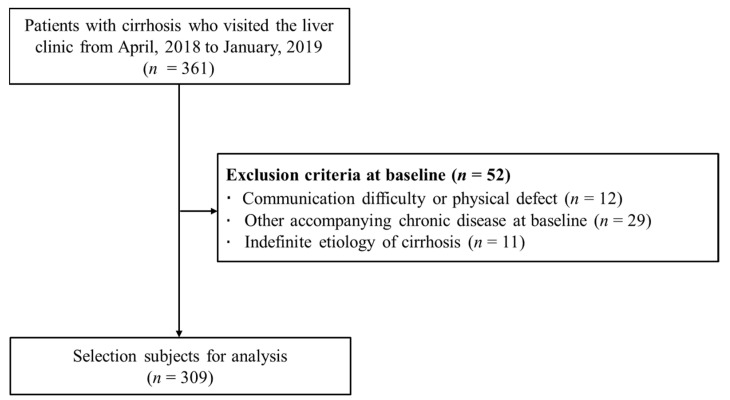
Flowchart of the participant selection process.

This study retrospectively evaluated 361 patients with cirrhosis who visited Hanyang University Hospital liver clinic between April 2018 and January 2019. Of the 361 patients, 12 patients were excluded from the analysis due to communication difficulties, and 29 patients were excluded because they had comorbid chronic conditions, including thyroid disease, chronic obstructive pulmonary disease, kidney disease, and cancer other than liver cancer. Additionally, 11 patients with inadequate food diary data were excluded (Figure 1), and a total of 309 patients were included as a result. The study was approved by the Institutional Review Board (IRB) of Hanyang University Hospital (IRB approval number: 2019-05-018-001), and the study was performed in accordance with the relevant guidelines. The requirement for obtaining informed consent was waived by the IRB.

In order to improve the quality of life and prevent nutrition-related medical complications, patients diagnosed with chronic liver disease should immediately assess and support their nutritional status through appropriate dietary interventions. In patients with chronic liver disease including liver cirrhosis, the importance of nutritional intake including protein has emerged. Therefore, in our hospital, we conducted a dietary intake evaluation using FFQ to check and educate patients with chronic liver disease. A study of retrospective design was conducted based on the results obtained.

### 2.2. Inclusion and Exclusion Criteria

Patients aged ≥19 years with cirrhosis were included in the study. LC was diagnosed based on clinical judgment or the results of imaging studies. Patients were classified as having alcoholic LC, non-alcoholic steatohepatitis (NASH-LC), or viral hepatitis (viral LC) according to the etiology of liver disease.

Patients with a history of medication usage and dietary interventions to control weight within the last 6 months, a comorbid chronic condition that may cause weight loss (thyroid disease, chronic obstructive pulmonary disease, or kidney disease), and malignancy other than liver cancer were excluded.

### 2.3. Quality and Quantity of Muscle Mass

To diagnose sarcopenia, muscle mass was measured using bioelectrical impedance analysis (BIA) (Inbody 370; Inbody USA, Cerritos, CA, USA). Sarcopenia was defined as the volume of appendicular skeletal muscle (ASM) divided by height in meters squared (m^2^). The cutoff values were 7.0 kg/m^2^ for men and 5.7 kg/m^2^ for women [21]. Fat-free mass index (FFMI) was computed as the volume of fat-free mass (kg), measured using BIA, divided by height in meters squared (m^2^). Handgrip tests were performed using a hand dynamometer (Jamar Hydraulic Hand Dynamometer; Asimow Engineering Co., Grass Valley, CA, USA) while the patients were in the standing position with their shoulders in full extension. The test was performed three times using the dominant hand, and the highest score was used in the analysis. Based on the Asian Working Group of Sarcopenia guidelines, 26 kg and 18 kg were used as the cutoff values for men and women, respectively [21]. 

### 2.4. Subjective Global Assessment

A clinical dietician with >5 years of clinical experience performed subjective global assessment (SGA) [22] via a survey and physical examination. The patients were assessed for weight loss, volume of dietary intake, gastrointestinal symptoms, functional disorders and subcutaneous fat loss, muscle atrophy, edema, and ascites. The patients were categorized into three groups: well-nourished (SGA A), mild/moderately malnourished (SGA B), and severely malnourished (SGA C) groups [22]. Patients categorized into the SGA B or C groups (SGA scores of ≥ 6) were screened for malnutrition.

### 2.5. Definition and Assessment of Dietary Intake

The nutritionist assessed the patients’ dietary intake using a food frequency questionnaire (FFQ) via a face-to-face interview. The nutrients obtained from various dietary sources were computed using CAN-Pro 4.0 [14] based on the data of the 6th Korea National Health and Nutrition Examination Survey (2013–2015) [15]. The estimated daily requirements were calculated using Schofield’s modification of the Harris–Benedict equation [23,24], and patients were screened for malnutrition if their total daily caloric consumption was lower than the estimated daily requirement.

### 2.6. Definition of Malnutrition

Patients with malnutrition were assessed for undernutrition using one of the following screening methods: diagnosing sarcopenia, use of nutritional assessment tools, or use of dietary intake journals [8]. According to the European Society for Clinical Nutrition and Metabolism guidelines, malnutrition is defined as BMI <18.5 kg/m^2^; unintentional weight loss exceeding 10% regardless of the time or weight loss of 5% within 3 months in addition to BMI <20 kg/m^2^ for individuals aged <70 years and a BMI of 22 kg/m^2^ for individuals aged ≥70 years; or FFMI <15 for women and <17 for men [5].

### 2.7. Statistical Analysis

Data analysis was performed using SPSS for Windows (Version 24; SPSS Inc., Chicago, IL, USA). All measurements are expressed as mean ± standard deviation. Analysis of variance, Student’s t-tests, and chi-square tests were used to examine the differences among groups, and a *p*-value of <0.05 was considered significant. Additionally, Cohen’s kappa analysis was performed to examine the level of agreement of malnutrition determined using the different malnutrition assessment methods. This study was a descriptive study based on multiple patient charts and did not include calculation of the sample size. All patients with cirrhosis who visited the outpatient department during the study period were enrolled. 

## 3. Results

### 3.1. Basic Characteristics and Prevalence of Malnutrition 

A total of 309 patients with cirrhosis were included in the analyses. The mean patient age was 58.7 years, and 61.8% patients were men. In total, 88, 33, 172, and 16 patients were categorized into the alcoholic LC, NASH-LC, viral LC, and other LC groups, respectively. The prevalence of sarcopenia was 22.7% (70/309). The prevalence of malnutrition according to SGA was 11.7% (36/309), and the prevalence of inadequate dietary intake was 27.5% (85/309). Approximately 46.3% (*n* = 143) patients satisfied one of the three definitions of malnutrition (Table 1).

### 3.2. Prevalence of Malnutrition According to Etiology

**Figure 2:**
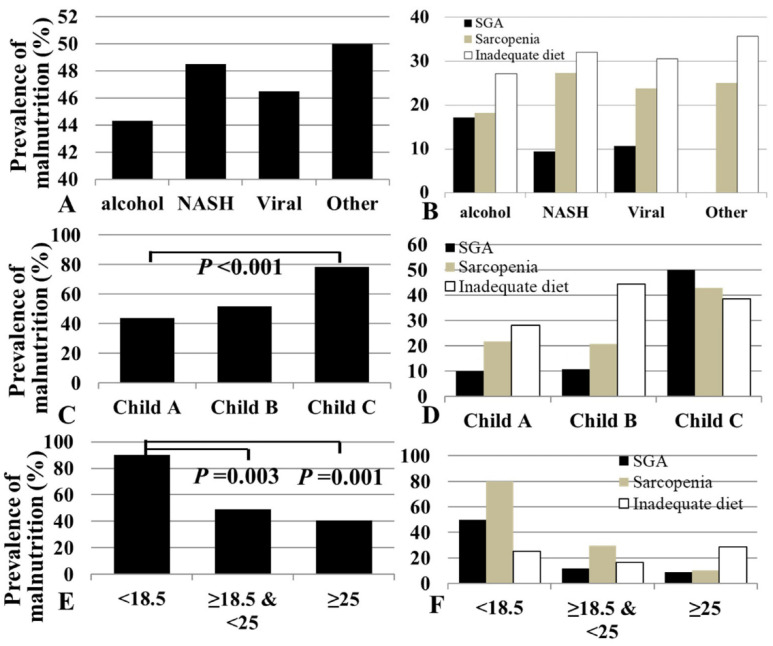
Prevalence of malnutrition defined based on the assessment results of different evaluation methods. (**A**) The prevalence of malnutrition according to the etiology of cirrhosis. (**B**) The prevalence of malnutrition according to the SGA, sarcopenia status, and dietary intake by etiology of cirrhosis. (**C**) The prevalence of malnutrition according to disease severity. (**D**) The prevalence of malnutrition according to the SGA findings, sarcopenia status, and dietary intake by disease severity. (**E**) The prevalence of malnutrition according to one of the definitions of malnutrition based on the body mass index. (**F**) The prevalence of malnutrition according to the SGA results, sarcopenia status, and dietary intake by body mass index.

In total, 39 (44.3%) patients in the alcoholic LC group, 16 (48.5%) patients in the NASH-LC group, 80 (46.5%) patients in the viral LC group, and 8 (50.0%) patients in the other LC group satisfied one of the three definitions of malnutrition (Table 1, Figure 2). Although the prevalence of decompensated cirrhosis (Child–Pugh B and C grades) and the model for end-stage liver disease (MELD) scores were higher in the alcoholic LC group than in the viral LC and NASH-LC groups (*p* = 0.005 and *p* < 0.001, respectively), the prevalence of malnutrition was not significantly different among these groups (*p* = 0.962; Table 1). The prevalence of sarcopenia, inadequate dietary intake, and SGA malnutrition was not significantly different according to the etiology of cirrhosis. Although the total energy intake was similar across the etiology-based groups, the alcoholic LC group had the highest protein and fat consumption. Lipid intake was higher in the alcoholic LC and NASH-LC groups. None of the groups showed other between-group differences in dietary intake. 

### 3.3. Prevalence of Malnutrition According to Disease Severity

In total, 266 (88.1%) patients were classified as having Child–Pugh grade A, while 43 (13.9%) patients were classified as having Child–Pugh grade B or C. A total of 117 (44.0%) in the Child–Pugh A group, 15 (51.7%) in the Child–Pugh B group, and 11 (78.6%) in the Child–Pugh C group satisfied one of the three definitions of malnutrition. The prevalence of malnutrition significantly increased with the increasing disease severity (*p* = 0.034; Table 2).

### 3.4. Prevalence of Malnutrition According to Body Mass Index

In total, 9 (90.0%) patients in the BMI <18.5 kg/m^2^ group, 81 (49.1%) patients in the BMI 18.5–25 kg/m^2^ group, and 52 (40.6%) patients in the BMI >25 kg/m^2^ group satisfied one of the three definitions of malnutrition. The prevalence of malnutrition increased with the decreasing BMI, and the differences were statistically significant (*p* = 0.007; Table 2). The proportion of patients with sarcopenia and low SGA in the BMI <18.5 kg/m^2^ group was 80% and 50%, respectively. The prevalence of sarcopenia increased with the decreasing BMI (*p* for trend <0.001).

### 3.5. Prevalence of Malnutrition According to Sarcopenia, SGA, and Dietary Intake

**Figure 3:**
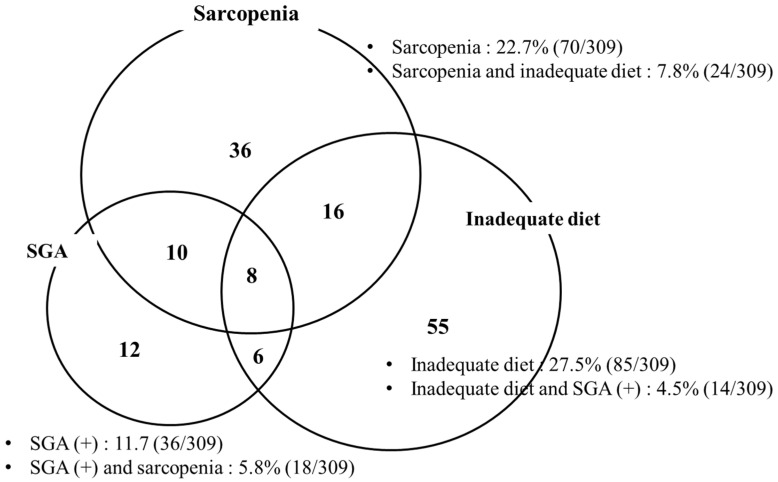
Degree of agreement between the definitions of malnutrition. Eighteen patients were diagnosed with malnutrition based on the sarcopenia status and SGA results, while 24 patients were diagnosed with malnutrition based on the sarcopenia status and dietary intake. Fourteen patients were diagnosed with malnutrition based on SGA results and dietary intake, and only eight patients fulfilled all the criteria for diagnosing malnutrition (sarcopenia, SGA, and dietary intake). The agreement (Cohen’s kappa value) among the sarcopenia, SGA, and energy intake-based methods was <0.217.

A total of 36 (13.6%), 70 (22.7%), and 85 (27.5%) patients had abnormal SGA, sarcopenia, and inadequate dietary intake, respectively (Table 3 and Figure 3). The prevalence of malnutrition by SGA significantly increased with the decreasing BMI and increasing Child–Pugh score. Malnutrition according to sarcopenia was significantly associated with BMI, but it was not statistically associated with the cause or severity of the disease. The prevalence of energy malnutrition (inadequate dietary intake) was not associated with the cause and severity of the disease or BMI.

### 3.6. Risk Factors for Malnutrition (Multivariate Analysis)

**Figure 4:**
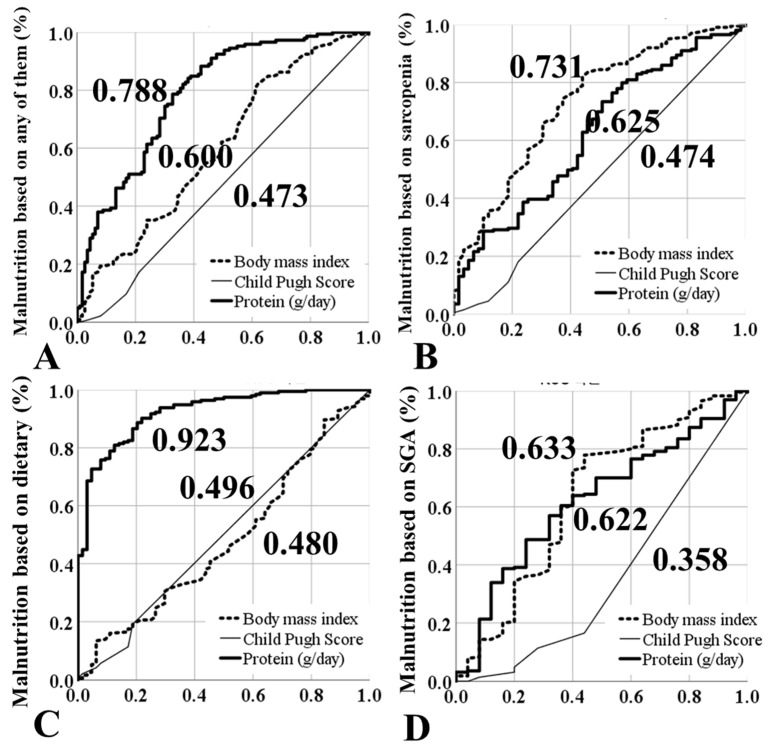
Receiver operating characteristic curve of factors affecting malnutrition. (**A**) Area under the curve of the factors affecting malnutrition based on the definition of malnutrition. (**B**) Area under the curve of factors affecting malnutrition based on sarcopenia. (**C**) Area under the curve of factors affecting malnutrition based on dietary intake. The estimated daily requirements were calculated using Schofield’s modification of the Harris–Benedict equation, and patients were screened for malnutrition if their total daily caloric consumption was lower than the estimated daily requirement. (**D**) Area under the curve of factors affecting malnutrition based on SGA.

Logistic regression analysis was performed to identify the risk factors that affect the prevalence of malnutrition. Age, sex, BMI, etiology, protein intake, Child-Pugh classification, and MELD score were used as parameters thought to affect malnutrition for univariate analysis. The etiology of LC did not influence the prevalence of malnutrition. However, low protein intake (<1.0 g/kg), Child–Pugh C grade, older age, and low BMI were independent risk factors for malnutrition (Table 4). Among the patients with cirrhosis, 43.6% patients consumed <1.0 g/kg of protein per day. The prevalence of malnutrition was 69.2% among patients with a protein intake of <1.0 g/kg/day. Based on the receiver operating characteristic curve, protein intake showed the best performance in predicting malnutrition (Figure 4). The areas under the curve for protein intake, BMI, and the MELD score were 0.788, 0.600, and 0.473, respectively.

## 4. Discussion

In this study, 46.3% patients had malnutrition. The prevalence rates of malnutrition were 78.6% and 64.4% among patients with Child–Pugh grade C and those with protein intake <1.0 g/kg/day, respectively.

A previous study reported a protein intake of 1.16–1.31 g/kg/day in patients with liver disease, which is not significantly different from the 1.29 g/kg/day protein intake indicated in this study. In a previous study, the average patient age with compensated viral liver cirrhosis was 68.3 years [20], which was higher than that in this study (58.7 years). However, the mean patient age in this study was similar to that in other studies, and no significant difference was observed in terms of sex. Previous studies included non-cirrhotic Hepatitis C virus (HCV) patients [18], non-LC and LC patients [19], or viral LC patients [20]. This study included all patients with chronic liver disease and analyzed and compared the nutritional intake according to the LC status and cause and severity of the disease.

A 24-h recall method, a food intake frequency recall method, a meal diary method, and an actual measurement method were used to determine the study participants’ nutrition intake. In most previous studies, protein intake was assessed using the 24-h recall method. The recall method is used to estimate the nutrient intake from the surveyed data based on the type and amount of food consumed within 24 h. It can be performed within a short period of time, and only slight changes in the dietary habits can occur; however, this method cannot be used to measure the food intake based on the 24-h data, and a recall bias may potentially occur. The most accurate measurement method is the weighing method, which can accurately measure the food intake by weighing the food ingredients cooked before meals and subtracting the amount of food remaining after the meal. However, this method is difficult to apply in a clinical setting. This study confirmed the nutrient intake in patients with chronic liver disease using the dietary diary method. The results of assessment using this method were not considered valid due to the limited food list. However, it had lesser recall bias and was a relatively accurate method, as it was possible to record the type of food and food intake in a diary format while the participant was eating.

In this study, 13.6% patients with cirrhosis had malnutrition based on the SGA results. In previous studies, the prevalence of malnutrition varied from 5% to 99% according to the definition of malnutrition [3,9,10,11,19,25]. In a previous study involving 1402 patients published in 1994, mid-arm muscle circumference and mid-arm fat circumference were measured, and malnutrition was defined as a median value of <5%. In this study, the prevalence of malnutrition was 30%. The prevalence of malnutrition was high in patients with Child–Pugh grades B and C, and no significant difference was observed between the two study groups according to the cause of cirrhosis [11]. Malnutrition was defined as protein-calorie malnutrition in 300 patients, and 38.3% malnutrition cases were reported in 2006. The prevalence of malnutrition also increased with the increasing disease severity, but malnutrition was not found to be related with the cause and prevalence of cirrhosis [19]. Other previous studies have used various evaluation methods. SGA, prognostic nutritional index, and handgrip strength were used to diagnose malnutrition in 50 patients in 2005, and 28%, 18.7%, and 63% of the patients who underwent the abovementioned tests, respectively, were reported to have malnutrition [25]. Another study diagnosed malnutrition according to handgrip strength, mid-arm muscle circumference, SGA, and corrected BMI and reported prevalence rates of 67%, 58%, 58%, and 5%, respectively [3]. However, no recent studies have used these evaluation methods in a large number of patients who showed an improvement in nutritional status compared with that before 2000. This study was conducted to evaluate the prevalence of malnutrition using the anthropometric method, SGA, and dietary intake in >300 patients with cirrhosis.

It is unclear whether there is a difference in the prevalence of malnutrition according to the etiology of cirrhosis. Some studies showed a higher prevalence of malnutrition in patients with alcoholic cirrhosis than in those with non-alcoholic cirrhosis [26,27]. However, a significant difference was found in the baseline severity of liver disease between the two patient groups, and the assessment method used in these studies may not be optimal. For example, it might be inappropriate to measure the simple skin fold thickness and body fat mass of patients with NASH-associated cirrhosis to assess malnutrition. The total fat mass is relatively preserved in patients with NASH cirrhosis. In our study and previous studies [3,28], the prevalence of low SGA and sarcopenia did not differ according to etiology of the disease. 

The present study has several limitations. First, sarcopenia was diagnosed by measuring the ASM using BIA, and the results could be influenced by excess body fluid. Although the proportion of patients with generalized edema and/or ascites was small, the prevalence of sarcopenia can be overestimated in patients with decompensated diseases. Assessment of the psoas muscle area using abdominal computed tomography, the phase angle α, or body cell mass, which is not affected by fluid accumulation, is more appropriate in patients with decompensated cirrhosis to evaluate the presence of sarcopenia [29,30]. Second, the study included outpatients, and the number of patients with decompensated cirrhosis was relatively small. It would be difficult to apply to patients other than Child A. Hence, future studies should be conducted in a larger sample of patients with cirrhosis to assess their nutritional status and evaluate the prevalence of malnutrition in terms of the severity of cirrhosis. Third, nutrient intake assessments were performed using an FFQ. The volume of food intake is more accurately evaluated using a 3-day dietary journal, which includes the food intake during the weekend. In the nutritional evaluation of patients with chronic liver disease, a 3-day meal record is used, but due to difficulties in collecting data, a 24-h recall can also be used. When comparing FFQ and 24 h recall, there is a study that the FFQ method has a drawback that it may be somewhat underestimated. However, it is known that FFQ can also be used in a reliable way through validation studies conducted in comparison with the 24 h recall method. Although the FFQ allows the examination of dietary habits in patients with chronic disease, it is difficult to accurately assess the volume of food intake using this method.

## 5. Conclusions

In conclusion, the prevalence of malnutrition, assessed using various assessments, was 46.3%. The prevalence of malnutrition increased as the disease severity increased and protein consumption decreased. The prevalence of malnutrition was extremely high in patients with a protein intake of <1.0 g/kg. Taken together, the study suggests that protein intake is a good indicator of adequate dietary intake.

## Figures and Tables

**Table 1:** General characteristics and nutritional assessment of the patients based on etiology.

Characteristics	Total LC (*n* = 309)	Alcoholic LC (*n* = 88)	NASH-LC (*n* = 33)	Viral LC (*n* = 172)	Other LC (*n* = 16)	*p*
Age (yr.)	58.7 ± 9.26	56.7 ± 8.94	63.4 ± 9.31	58.5 ± 9.34	59.3 ± 6.78	0.005
Sex (%)						
Male	191 (61.8)	82 (93.2)	12 (36.4)	93 (54.1)	4 (25.0)	<0.001
Female	118 (38.2)	6 (6.8)	21 (63.6)	79 (45.9)	12 (75.0)	
Height (cm)	164.5 ± 8.63	168.3 ± 6.66	161.4 ± 8.33	164.0 ± 8.84	156.5 ± 7.32	<0.001
Weight (kg)	66.6 ± 12.12	68.0 ± 12.80	63.7 ± 10.53	66.9 ± 12.25	61.5 ± 8.28	0.119
BMI (%)						0.303
<18.5 (kg/m^2^)	10 (3.3)	4 (4.8)	1 (3.0)	5 (2.9)	0 (0)	
18.5–25 (kg/m^2^)	165 (54.5)	52 (61.9)	18 (54.6)	85 (50.0)	10 (62.5)	
≥25 (kg/m^2^)	128 (42.2)	28 (33.3)	14 (42.4)	80 (47.1)	6 (37.5)	
ASM (kg)	20.1 ± 4.79	22.4 ± 4.19	18.2 ± 4.72	19.7 ± 4.77	16.3 ± 2.37	<0.001
Percent body fat (%)	28.0 ± 9.12	21.8 ± 8.61	31.1 ± 8.37	29.8 ± 8.17	34.4 ± 6.59	<0.001
FFMI (kg/m^2^)	17.5 ± 2.41	18.6 ± 2.37	16.7 ± 2.31	17.2 ± 2.36	16.4 ± 1.59	<0.001
Handgrip strength (kg)	30.0 ± 9.89	33.6 ± 7.75	23.9 ± 8.03	29.7 ± 10.68	25.1 ± 7.30	<0.001
Sarcopenia (%)	70 (22.7)	16 (18.2)	9 (27.3)	41 (23.8)	4 (25.0)	0.339
Child–Pugh (%)						0.005
A	266 (86.1)	66 (75.0)	30 (90.9)	155 (90.1)	15 (93.8)	
B	29 (9.4)	14 (15.9)	2 (6.1)	12 (7.0)	1 (6.2)	
C	14 (4.5)	8 (9.1)	1 (3.0)	5 (2.9)	0 (0)	
MELD score	8.6 ± 2.72	9.8 ± 3.78	8.2 ± 2.82	8.2 ± 1.84	7.28 ± 1.05	<0.001
SGA (%)						0.186
A	267 (86.4)	72 (82.8)	29 (90.6)	151 (89.3)	15 (100.0)	
B	34 (11.0)	14 (16.1)	3 (9.4)	17 (10.1)	0 (0)	
C	2 (0.7)	1 (1.1)	0 (0.0)	1 (0.6)	0 (0)	
Dietary intake						
Total energy (kcal)	2090 ± 910	2294 ± 940	2069 ± 962	2014 ± 895	1821 ± 632	0.114
Inadequate (%)	85 (27.5)	22 (27.2)	9 (32.1)	49 (30.6)	5 (35.7)	0.894
Carbohydrate (g)	310 ± 126	316 ± 131	310 ± 130	310 ± 125	281 ± 99	0.843
Protein (g)	86 ± 46	100 ± 51	87 ± 48	80 ± 42	74 ± 39	0.024
Lipid (g)	57 ± 35	66 ± 38	57 ± 37	53 ± 32	48 ± 29	0.038
Carbohydrate (g/kg)	4.8 ± 1.88	4.8 ± 2.07	4.8 ± 2.07	4.7 ± 1.76	5.0 ± 1.79	0.869
Protein (g/kg)	1.3 ± 0.68	1.5 ± 0.79	1.3 ± 0.70	1.2 ± 0.60	1.3 ± 0.64	0.030
Lipid (g/kg)	0.8 ± 0.45	1.0 ± 0.67	0.8 ± 0.50	0.8 ± 0.46	0.8 ± 0.45	0.044
Cholesterol	417 ± 277	417 ± 316	438 ± 246	402 ± 231	520 ± 480	0.448
Vitamin A (µg)	984 ± 562	954 ± 596	1023 ± 575	965 ± 477	1254 ± 986	0.264
Vitamin C (mg)	145 ± 94	151 ± 113	130 ± 74	141 ± 81	187 ± 124	0.243
Vitamin D (µg)	5.0 ± 4.47	4.7 ± 3.77	6.4 ± 7.15	4.7 ± 3.56	7.4 ± 7.74	0.040
Vitamin E (mg)	18.2 ± 10.46	17.2 ± 9.78	20.1 ± 11.03	17.7 ± 9.54	24.5 ± 17.85	0.058
Thiamin (mg)	1.6 ± 0.84	1.5 ± 0.79	1.9 ± 1.10	1.6 ± 0.77	1.9 ± 1.14	0.113
Riboflavin (mg)	1.4 ± 0.78	1.4 ± 0.77	1.6 ± 0.88	1.4 ± 0.66	1.9 ± 1.38	0.088
Calcium (mg)	599 ± 356	584 ± 404	649 ± 261	579 ± 305	788 ± 608	0.146
Iron (mg)	12.9 ± 6.51	12.0 ± 6.65	14.4 ± 6.70	12.7 ± 5.79	15.7 ± 10.62	0.125
Malnutrition (%)						
by ESPEN CPG	36 (11.6)	11 (13.3)	2 (6.1)	18 (10.7)	2 (12.5)	0.731
by EASL CPG	143 (46.3)	39 (44.3)	16 (48.5)	80 (46.5)	8 (50.0)	0.962

Data are expressed as mean ± standard deviation (number, %). Alcoholic LC, alcoholic liver cirrhosis; NASH-LC, nonalcoholic steatohepatitis-related liver cirrhosis; viral LC, viral liver cirrhosis; BMI, body mass index; ASM, appendicular skeletal muscle; FFMI, fat-free mass index; MELD, model for end-stage liver disease; SGA, subjective global assessment; EASL CPG, European Association for the Study of the Liver Clinical Practice Guidelines; ESPEN CPG, European Society for Parenteral and Enteral Nutrition Clinical Practice Guidelines.

**Table 2:** Clinical parameters of malnutrition in patients classified based on liver function and body mass index.

	Child–Pugh Classification				Body Mass Index (kg/m^2^)			
Characteristics	A (*n* = 266)	B (*n* = 29)	C (*n* = 14)	*p*	<18.5 (*n* = 10)	18.5–25 (*n* = 165)	≥25 (*n* = 128)	*p*
BMI (%, kg/m^2^)				0.145				
<18.5	8 (3.1)	0 (0.0)	2 (14.3)					
18.5–25	144 (55.4)	15 (51.7)	6 (42.8)					
≥25	108 (41.5)	14 (48.3)	6 (42.8)					
Child–Pugh classification (%)								0.145
A					8 (80.0)	144 (87.3)	108 (79.5)	
B					0 (0.0)	15 (9.1)	14 (13.7)	
C					2 (20.0)	6 (3.3)	6 (6.8)	
ASM (kg)	20 ± 4.7	21 ± 4.4	23 ± 5.8	0.041	16 ± 3.6	19 ± 4.4	22 ± 4.9	<0.001
FFMI (kg/m^2^)	17 ± 2.3	21 ± 4.5	23 ± 5.8	<0.001	14 ± 1.7	17 ± 2.0	19 ± 2.3	<0.001
Handgrip strength(kg)	30 ± 10.2	31 ± 8.3	28 ± 4.8	0.637	23 ± 6.0	29 ± 9.9	32 ± 9.6	0.006
Sarcopenia (%)	58 (21.8)	6 (20.7)	6 (42.9)	0.160	8 (80.0)	49 (29.7)	13 (10.2)	<0.001
SGA (%)				<0.001				<0.001
A	235 (90.0)	25 (89.3)	7 (50.0)		5 (50.0)	146 (88.5)	112 (91.1)	
B	24 (9.2)	3 (10.7)	7 (50.0)		4 (40.0)	18 (10.9)	11 (8.9)	
C	2 (0.8)	0 (0.0)	0 (0.0)		1 (10.0)	1 (0.6)	0 (0.0)	
Dietary intake								
Total energy (kcal)	2086 ± 900	2099 ± 969	2137 ± 1067	0.982	1782 ± 504	2015 ± 888	2217 ± 938	0.151
Inadequate (%)	68 (28.0)	12 (44.4)	5 (38.5)	0.167	2 (25.0)	44 (16.3)	37 (28.6)	0.797
Carbohydrate (g)	309 ± 124	315 ± 120	335 ± 169	0.763	265 ± 73	300 ± 121	331 ± 131	0.095
Protein (g)	86 ± 45	88 ± 58	84 ± 51	0.969	71 ± 26	84 ± 46	90 ± 45	0.377
Lipid (g)	57 ± 34	57 ± 41	53 ± 33	0.939	48 ± 20	54 ± 34	60 ± 35	0.337
Malnutrition (%)								
by ESPEN CPG	27 (10.5)	2 (6.9)	4 (28.6)	0.083	10 (100.0)	21 (13.0)	2 (1.6)	<0.001
by EASL CPG	117 (44.0)	15 (51.7)	11 (78.6)	0.034	9 (90.0)	81 (49.1)	52 (40.6)	0.007

Data are expressed as mean ± standard deviation (number, %). BMI, body mass index; ASM, appendicular skeletal muscle mass; FFMI, fat-free mass index; SGA, subjective global assessment; EASL CPG, European Association for the Study of the Liver Clinical Practice Guidelines; ESPEN CPG, European Society for Parenteral and Enteral Nutrition Clinical Practice Guidelines.

**Table 3:** Differences in liver function, body mass index, and etiology according to status of malnutrition.

Classification	Sarcopenia		SGA		Inadequate Diet		Any	
	Presence (*n* = 70)	*p*	Presence (*n* = 36)	*p*	Presence (*n* = 85)	*p*	Presence (*n* = 143)	*p*
BMI (%, kg/m^2^)		<0.001		0.017		0.521		0.007
<18.5	8 (80.0)		7 (70.0)		2 (20.0)		9 (90.0)	
18.5–25	49 (29.7)		18 (10.9)		44 (26.7)		81 (49.1)	
≥25	13 (10.2)		11 (8.6)		37 (28.9)		52 (40.6)	
Child–Pugh (%)		0.160		<0.001		0.111		0.034
A	58 (21.8)		25 (8.8)		68 (25.6)		117 (44.0)	
B	6 (20.7)		4 (26.7)		12 (41.4)		15 (51.7)	
C	6 (42.9)		7 (70.0)		5 (35.7)		11 (78.6)	
Etiology (%)		0.339		0.055		0.510		0.962
Alcohol	16 (18.2)		15 (17.0)		22 (25.0)		39 (44.3)	
NASH	9 (27.3)		3 (9.1)		9 (27.3)		16 (48.5)	
Viral	41 (23.8)		18 (10.5)		49 (28.5)		80 (46.5)	
Others	4 (25.0)		0 (0.0)		5 (31.3)		8 (50.0)	

BMI, body mass index; SGA, subjective global assessment; NASH, nonalcoholic steatohepatitis; *p* < 0.05.

**Table 4:** Risk factors of malnutrition.

	Univariate		Multivariate	
	Exp(ß)	*p*	Exp(ß)	*p*
Age	1.03	0.009	1.03	0.011
BMI (kg/m^2^)	0.90	0.002	0.84	0.002
Etiology		0.691		
Protein (g)	0.22	<0.001	0.18	<0.001
Child–Pugh classification		0.017		

Data are expressed as *p* values and Exp(ß). Exp(ß), odds ratio confidence interval; BMI, body mass index; *p* < 0.05.

## Data Availability

The data presented in this study are available on request from the corresponding author. The data are not publicly available due to privacy of patients.
